# Effects of esketamine infusion on tourniquet-induced hypertension in patients undergoing below-knee orthopedic surgery: protocol for a randomized controlled trial

**DOI:** 10.1080/07853890.2025.2478479

**Published:** 2025-03-13

**Authors:** Kai Jiang, Wen-qian Zhao, Yao-yu Ying, Min-yuan Zhuang, Hai-jing Shi, Fu-hai Ji, Ke Peng

**Affiliations:** ^a^ Department of Anesthesiology, First Affiliated Hospital of Soochow University, Suzhou, Jiangsu, China; ^b^ Institute of Anesthesiology, Soochow University, Suzhou, Jiangsu, China; ^c^ Department of Rheumatology, Children’s Hospital of Soochow University, Suzhou, Jiangsu, China; ^d^ Department of Medical affairs, The Second Affiliated Hospital of Soochow University, Suzhou, China

**Keywords:** Esketamine, tourniquet-induced hypertension, below-knee orthopedic surgery, randomized controlled trial, nerve block, quality of recovery

## Abstract

**Introduction:**

Application of a tourniquet reduces surgical bleeding while causing pain and tourniquet-induced hypertension (TIH). Deeper anesthesia and additional opioids are often insufficient to mitigate TIH but are associated with prolonged recovery and complications. Herein, we describe the protocol for a clinical trial investigating whether intraoperative esketamine infusion would reduce the rate of TIH during below-knee orthopedic surgery.

**Patients and Methods:**

This prospective, randomized, double-blind, controlled trial will include a total of 80 adults scheduled for below-knee orthopedic surgery under general anesthesia. Patients will be randomly assigned in a 1:1 ratio to receive either esketamine infusion at 0.25 mg/kg/h or normal saline infusion after anesthesia induction and during tourniquet inflation. Both groups will receive ultrasound-guided unilateral popliteal sciatic nerve block and adductor canal block with 0.25% ropivacaine. The primary outcome is the occurrence of TIH, defined as an increase in systolic blood pressure >30% of baseline during tourniquet inflation. Secondary outcomes include the incidence of hypotension during surgery; intraoperative hemodynamic changes; intraoperative opioid dose and total amount of esmolol; postoperative nausea and vomiting, dizziness, and mental side effects; postoperative pain at 1, 6, 12, and 48 h after surgery; postoperative analgesic consumption within 48 h after surgery; and length of postoperative hospital stay. Additionally, we will assess patients’ quality of recovery at 24 h after surgery.

**Discussion:**

This trial will determine the effects of esketamine infusion on TIH in patients who undergo below-knee orthopedic surgery under general anesthesia. Our results will offer a new insight into optimizing anesthetic care for surgical patients receiving tourniquet.

**Trial registration:**

Chinese Clinical Trial Registry (ChiCTR2400081237)

## Introduction

Tourniquet is often used to reduce surgical bleeding and improve surgical field during limb surgery. However, the application of tourniquet can result in severe pain and hemodynamic fluctuations [1]. Tourniquet-induced hypertension (TIH), usually defined as an increase in systolic blood pressure (SBP) more than 30% of baseline during tourniquet inflation, affects about 50%–90% of patients undergoing lower limb surgery [2–5]. Additional opioids and deeper anesthesia are often insufficient to inhibit TIH but are associated with other problems such as prolonged recovery, increased medical costs, and postoperative complications.

Studies have investigated pharmacological treatments and nerve blocks to reduce the occurrence of TIH. These interventions include ketamine, lidocaine, dexmedetomidine, and femoral nerve block [4–7]. Regarding the mechanisms of TIH, it is suggested that tourniquet inflation led to repeated nociceptive C-fiber afferent input and central sensitization through the activation of N-methyl-D-aspartate (NMDA) receptors [8,9]. Ketamine is an NMDA receptor antagonist that may block the tourniquet-induced responses. Esketamine is a dextrorotatory isomer of racemic ketamine and has four times greater affinity for the NMDA receptors than its stereoisomer arketamine, providing more analgesia with less psychiatric side effects [10–12]. Nonetheless, no studies have reported the impact of esketamine on TIH in patients undergoing limb surgery.

We design this randomized controlled trial to determine the effects of esketamine infusion on the incidence of TIH in patients undergoing below-knee orthopedic surgery with general anesthesia. Our primary hypothesis is that the administration of esketamine compared to normal saline would reduce the TIH incidence in these patients. We will also observe postoperative pain outcomes, quality of recovery and adverse effects.

## Patients and methods

### Study design

This is a single-center, prospective, randomized, double-blind, controlled clinical trial with two parallel groups. We will conduct this trial at the First Affiliated Hospital of Soochow University which is a tertiary teaching hospital in eastern China. The institutional review board approved this trial on January 31, 2024 (approval No. 2024-026), and we registered this study at the Chinese Clinical Trial Registry on February 27, 2024 (registration identifier: ChiCTR2400081237).

**Figure 1.**
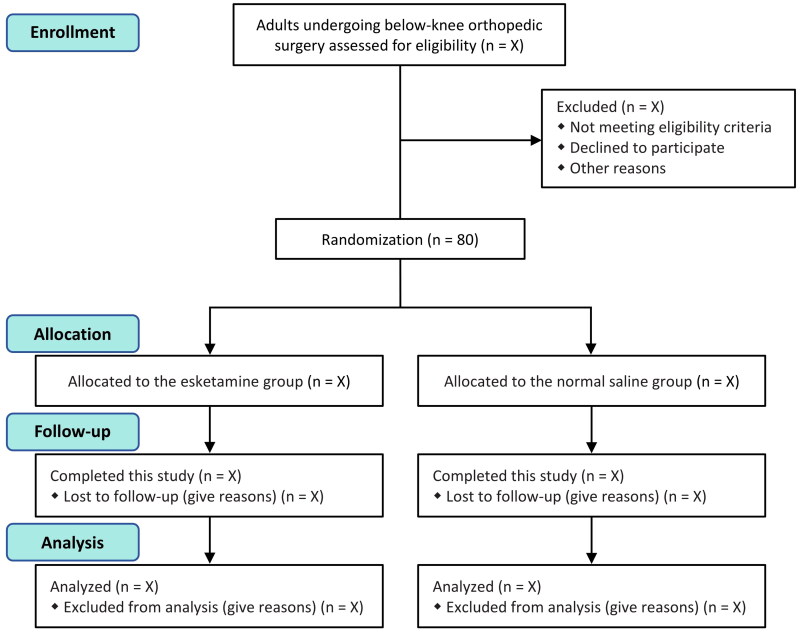
Study flowchart.

We plan to enroll a total of 80 patients between February and October 2024. We will conduct this trial based on the Declaration of Helsinki. All patients will provide their written inform consent. Figure 1 is the study flowchart. Table 1 shows the schedule of patient enrollment, interventions, and outcome assessment. This trial protocol conforms to the Standard Protocol Items: Recommendations for Interventional Trials (SPIRIT) guideline (Supplemental file 1) [13].

**Table 1.** Schedule of patient enrollment, interventions, and outcome assessment.

	Study period							
	Enrollment	Allocation	Post-allocation					Close-out
Timepoint	Preoperative visit	Before surgery	During surgery	1 h after surgery	6 h after surgery	12 h after surgery	48 h after surgery	Hospital discharge
**Enrollment**								
Inclusion criteria	**×**							
Exclusion criteria	**×**							
Written informed consent	**×**							
Baseline data	**×**							
Randomization		**×**						
Allocation		**×**						
**Interventions**								
Esketamine			**×**					
Normal saline			**×**					
**Study outcomes**								
Tourniquet-induced hypertension			**×**					
Hypotension			**×**					
Hemodynamic changes			**×**					
Opioid dose and antihypertensive therapy			**×**					
Adverse events				**×**	**×**	**×**	**×**	
Pain intensity				**×**	**×**	**×**	**×**	
Postoperative analgesic consumption							**×**	
Postoperative hospital stay								**×**

According to SPIRIT 2013 statement of defining standard protocol items for clinical trials.

### Inclusion and exclusion criteria

The inclusion criteria are: (1) aged 18–75 years; (2) American Society of Anaesthesiologists levels I to III; and (3) scheduled for unilateral below-knee orthopedic surgery (including surgery of the lower leg, ankle and foot) under general anesthesia.

### Exclusion criteria

The exclusion criteria are: (1) ischemic heart disease, uncontrolled hypertension, and cardiac, liver or renal insufficiency; (2) long-term use of opioids, sedatives, or antidepressants; (3) history of schizophrenia, epilepsy, Parkinson’s disease or myasthenia gravis; (4) contraindications of tourniquet (such as peripheral vascular disease or peripheral neuropathy); (5) estimated tourniquet inflation time <60 min or >150 min; (6) block site infection or allergy to local anesthetics; or (7) inability to communicate or refusal to participate in the study.

### Randomization and blinding

A research assistant not involved in the subsequent study performs the randomization with a ratio of 1:1 and blocks of 2, 4 and 6 using an online randomisation sequence tool (https://www.randomizer.org). To ensure concealment of allocation, this assistant prepares 80 sequentially numbered opaque envelopes. Each envelope contains a random code and the corresponding allocation information. When patients are admitted to the holding area, a research nurse who will not participate in perioperative care opens the envelopes one by one to assign patients into either an esketamine group or a normal saline group. This nurse is also responsible for preparing the study medications in identical 50-ml syringes, which will be delivered to the attending anesthesiologist. Both esketamine and normal saline are colorless and transparent solutions, so they cannot be visually distinguished. Patients, anesthesia and surgical team, outcome assessors, and a statistician will be blinded to the group assignment until the end of final analysis.

### Anaesthetic care and study interventions

Intraoperative monitoring includes electrocardiography, pulse oximetry, noninvasive blood pressure, nasopharyngeal temperature, and bispectral index (BIS). Patients will receive i.v. dexamethasone 5 mg, propofol 1.5–2 mg/kg, sufentanil 0.3 μg/kg, and rocuronium 0.6 mg/kg for anesthesia induction and tracheal intubation. Anesthesia will be maintained using 1–3% sevoflurane with 50% oxygen in air to achieve BIS values of 40–60. Mechanical ventilation will be started to maintain end-tidal carbon dioxide within 35–45 mmHg.

Before surgical incision, a tourniquet will be placed at the root of thigh on the surgical side. An elastic bandage will be used for exsanguination of the lower limb, and the tourniquet cuff will be inflated to 45 kPa (∼330 mmHg) according to our institutional standards. After anesthesia induction and during tourniquet inflation, patients in the esketamine group will receive a continuous infusion of esketamine at a rate of 0.25 mg/kg/h, while patients in the control group will receive normal saline infusion at an equivalent volume.

Sufentanil will be used only for induction and no other opioids will be given during surgery. For pain management, ultrasound-guided unilateral popliteal sciatic nerve block (using 0.25% ropivacaine 30 ml) and adductor canal block (using 0.25% ropivacaine 20 ml) will be performed after anesthesia induction and before surgery. Patients will receive i.v. palonosetron 0.25 mg at the end of surgery and i.v. flurbiprofen axetil 50 mg twice daily for two days or until hospital discharge. Rescue analgesia with i.v. tramadol 50–100 mg will be administered to treat postoperative pain with a numerical rating scale (NRS; 0–10, with 0 indicating no pain and 10 indicating the most severe pain) score ≥ 4.

Blood pressure will be measured using the upper-arm cuff method at 3-min intervals. The baseline SBP value is the average of three consecutive SBP measurements immediately before tourniquet inflation. In the case of SBP increase > 30% from baseline for at least two consecutive measurements, esmolol will be i.v. administered at a rate of 200 mg/h until SBP returns to normal (defined as increase < 20% above baseline). Hypotension (defined as an SBP decrease > 30% of baseline or SBP < 90 mmHg) will be treated with ephedrine 6 mg, and heart rate (HR) < 50 beats/min will be treated with atropine 0.5 mg. Other anesthetic care will be left to the discretion of the attending anesthesiologist.

### Outcome measures

The primary outcome of this trial is the occurrence of TIH, defined as an SBP increase > 30% of the baseline value at any time during tourniquet inflation, which is in line with that in the literature [2,3,5,14].

The secondary outcomes include: (1) the incidence of hypotension during surgery; (2) intraoperative changes in SBP, mean blood pressure, and HR; (3) intraoperative opioid dose and total amount of esmolol; (4) postoperative nausea and vomiting (PONV), dizziness, and mental side effects; (5) NRS pain scores at 1, 6, 12, and 48 h after surgery; (6) postoperative analgesic consumption within 48 h after surgery; and (7) length of postoperative hospital stay. Additionally, we will assess patients’ recovery quality at 24 h after surgery using the 15-item quality of recovery (QoR-15) scale [15].

### Data management

During a preoperative visit, a trial investigator will screen patients based on the eligibility criteria, and patient demographics and baseline data will be collected. The occurrence of TIH and the use of esmolol will be documented by the anesthesia team who are blinded to the group assignment. Surgical data, hemodynamic data and intraoperative doses of anesthetics and analgesics will be extracted from the electronic anesthesia system. During the postoperative follow-up, an investigator who does not participate in patient care will assess postoperative outcomes. Case report forms will be used for data collection, and then an electronic database will be established. The Ethics Committee will conduct an ongoing review of the trial implementation.

### Sample size estimation

According to recent studies, the incidence of TIH ranged from 56% to 93% in patients undergoing lower limb surgery under general anesthesia [2–5]. We assume that 66% of patients in the control group would develop TIH, and the infusion of esketamine would reduce the TIH incidence by 50% (i.e. an absolute reduction of 33%). Based on this assumption with a power of 80% at a significance level of 0.05, this trial needs 33 patients per group. Thus, we plan to enroll a total of 80 patients (*n* = 40 in each group) to account for possible dropouts. The sample size was calculated using PASS (version 11.0.7, NCSS, LLC, Kaysville, UT, USA).

### Statistical analysis

Normal distribution will be examined using the Shapiro-Wilk test. Continuous data will be presented as means and standard deviations (SDs) and compared using the unpaired t test if normally distributed, or as medians and interquartile ranges (IQRs) and compared using the Mann-Whitney test if nonnormally distributed. Categorical data will be reported as numbers (%) and compared using χ^2^ test or Fisher exact test.

Patient characteristics will be analyzed using the descriptive statistics only. The study outcomes will be analyzed using the relative risks or differences in means/medians with the 95% confidence intervals. For the primary outcome of TIH incidence, the treatment effect will be further analyzed using the number needed to treat. Subgroup analyses for the primary outcome are planned on the following subgroups, including sex (male vs. female), age (18–50 years vs. >50 years), history of hypertension (yes vs. no), and length of tourniquet inflation (60–90 min vs. >90 min). All analyses will be intention-to-treat, including all randomized patients with available data. No multiple comparison corrections are planned for the secondary outcomes. We will perform the statistical analyses using the SPSS (version 25.0, IBM, NY, USA). A two-sided *p* < 0.05 denotes a statistically significant difference.

## Discussion

In this randomized controlled trial, we will recruit a total of 80 patients undergoing below-knee orthopedic surgery to evaluate the effects of esketamine infusion vs. normal saline during tourniquet inflation on the occurrence of TIH. In addition, we will compare the two groups on the total amount of esmolol needed to treat TIH, intraoperative hypotension, postoperative pain, analgesic consumption, adverse effects, and postoperative quality of recovery. We will conduct this study following the Consolidated Standards of Reporting Trials guidelines [16].

Previous studies have suggested that a single injection of ketamine reduced the incidence of TIH [6,14]. However, neuropsychiatric symptoms often occur following ketamine administration, which limited its clinical use. Compared to ketamine, esketamine produces more analgesia and less side effects [12]. In a recent study, Wang et al. demonstrated that a single dose of esketamine 0.2 mg/kg infused over 40 min after childbirth reduced major depression at 42 days in mothers with symptoms of prenatal depression [17]. Neurological or mental side effects associated with esketamine depend on the rate of administration. Therefore, we will use a continuous infusion of esketamine at a rate of 0.25 mg/kg/h in our patients.

Opioids are regarded as the cornerstone of analgesia for patients undergoing surgery. However, use of opioids is associated with increased risks of adverse events such as PONV, hyperalgesia, respiratory depression, gastrointestinal paralysis, and delirium [18,19]. To reduce opioid-related side effects and avoid potential influence on intraoperative hemodynamics, sufentanil will be used only during the induction of anesthesia and no other opioids will be given throughout the surgery. Instead, we will apply ultrasound-guided popliteal sciatic nerve block and adductor canal block to improve analgesia and blunt the fluctuations of blood pressure.

This study has its limitations. First, the dose of esketamine infusion is based on the previous studies [10,20]; however, the optimal esketamine dose to mitigate TIH and improve postoperative outcomes still needs more studies. Next, it is our routine clinical practice that all patients will receive a tourniquet inflation pressure of 45 kPa which is higher than the pressure of 250–300 mmHg in the literature [2,3,5]. This difference may affect the incidence of TIH and the use of esmolol. Last, this is a single-center trial and future studies are warranted to validate our results at different institutions.

In summary, this randomized controlled trial will determine the effects of esketamine infusion on TIH in patients who undergo below-knee orthopedic surgery under general anesthesia. The results of this study will offer a new insight into optimizing anesthetic care for surgical patients receiving tourniquet.

## Supplementary Material

Supplementary file 1_SPIRIT checklist.doc

## Data Availability

The full protocol, participant-level dataset, statistical plan, and informed consent materials can be available *via* contacting the corresponding author after the formal publication of this trial.
